# A Small Molecule Selectively Targets N-Myc to Suppress Neuroblastoma Cancer Progression

**DOI:** 10.7150/ijbs.97195

**Published:** 2025-07-28

**Authors:** Ying Miao, Huang Chen, Yuzhan Li, Liting Li, Jiangnan Ye, Jingwen Zhang, Jiayu Wang, Haigang Wu, Guihong Li, Yihua Chen, Zhengfang Yi, Mingyao Liu

**Affiliations:** 1 Shanghai Key Laboratory of Regulatory Biology, Institute of Biomedical Sciences and School of Life Sciences, East China Normal University, Shanghai 200241, China.; 2 Changning Maternity and Infant Health Hospital, East China Normal University, 500 Dong Chuan Rd, Shanghai 200241, China.; 3 School of Pharmaceutical Sciences and Yunnan Key Laboratory of Pharmacology for Natural Products and Yunnan College of Modern Biomedical Industry, Kunming Medical University, Kunming, Yunnan 650500, China.; 4 Southern Medical University Affiliated Fengxian Hospital, 201499, Shanghai, China.

**Keywords:** Neuroblastoma, N-Myc, Small-molecule inhibitor, Threonine-58, Proteasome.

## Abstract

Neuroblastoma, a prevalent and lethal extracranial solid tumor in childhood, remains a significant challenge in pediatric oncology worldwide. High-risk neuroblastoma (HR-NB) is particularly aggressive and linked to a poor prognosis due to the limited availability of effective treatments. The aberrant amplification of the *MYCN* gene is a critical genetic alteration observed in neuroblastoma conferring poorer clinical outcomes. To date, no drugs targeting N-Myc have been approved. In this study, we successfully established a novel high-throughput screening system targeting N-Myc and identified the first small molecule inhibitor, N78, which exhibits selective, high affinity for N-Myc over c-Myc. N78 selectively degrades N-Myc, suppresses the expression of its target genes, and effectively diminishes the viability of *MYCN*-dependent tumor cells. Notably, N78 demonstrates acceptable tolerability and induces significantly enhanced tumor regression *in vivo* compared to Myci975, a leading candidate among c-Myc/N-Myc inhibitors. Mechanistically, N78 promotes the phosphorylation of N-Myc at threonine-58, leading to its degradation via the ubiquitin-proteasomal pathway. This study presents the first selective N-Myc inhibitor N78, and highlights the promise of small-molecule N-Myc inhibitors as both chemical probes and potential anti-cancer therapies for neuroblastoma.

## Introduction

Neuroblastoma, a sympathetic nervous system cancer originating from neural crest cells, is the most common extracranial solid tumor in childhood. It accounts for approximately 15% of all cancer-related deaths in the pediatric population 1-3. About half of these children are diagnosed with localized low-risk neuroblastoma, while the other half face metastatic high-risk neuroblastoma, which has a 5-year survival rate of less than 50% 1, 4. For cases of low-risk neuroblastoma, standard practice involves minimal therapeutic intervention, with a potential for complete cure via surgical removal alone or through spontaneous tumor regression 5, 6. Patients with intermediate-risk neuroblastoma undergo milder chemotherapy, and may also have residual tumor surgically removed 7. The current standard treatment for high-risk neuroblastoma includes a combination of high-dose cytostatics, radiation therapy, surgery, myeloablative therapy with stem cell reinfusion, long-term maintenance therapy with retinoic acid, and immunotherapy using anti-disialoganglioside 2 antibodies 2. Regrettably, despite adherence to the standard treatment protocols, about one-third of children with high-risk neuroblastoma show unsatisfactory responses. Furthermore, nearly half of high-risk patients either do not respond favorably to initial treatments or experience a relapse within two years. The variable outcomes of current treatments, ranging from complete tumor regression to the emergence of multi-drug resistance and severe toxicities, underscore the complexity of treating neuroblastoma 8.

MYC transcription factors are aberrantly activated in the majority of human cancers 9-11. Its isoforms (c-MYC (*MYC*), *MYCN*, and *MYCL*) are crucial in regulating transcription, metabolism, cell division and immune surveillance 12. Unlike c-Myc, which is altered in a variety of tumors, N-Myc primarily functions as a significant driver of pediatric malignancies, including neuroblastoma 10, 13. N-Myc is overexpressed in approximately 25% of neuroblastomas and is associated with aggressive disease progression and poor outcomes 14, emphasizing its potential as a valuable therapeutic target for N-Myc-dependent cancers 15, 16. However, developing N-Myc inhibitors have been proved challenging due to its disordered protein structure, absence of binding sites, and involvement of multiple co-regulatory factors 17-19. Strategies to overcome these challenges include targeting its transcriptional activity with Bromodomain and Extraterminal Domain (BET) family inhibitors, modulating epigenetic factors with histone deacetylase inhibitors, and promoting its proteasomal degradation 20-22. Despite these efforts, tumor cells have various means of evading these pathways, thereby sustaining the expression and activity of MYC-family proteins. This resilience stems from the complex signaling pathways that regulate MYC genes and proteins, coupled with the multifaceted functions of MYC 17, 23, 24. As a result, direct pharmacologic inhibition of Myc proteins might represent the most reliable strategy, as is the case with most transcription factors 25, 26. However, the task of developing direct inhibitors of N-Myc remains arduous, even after decades of research 27-29.

In this study, a high-throughput screening system designed specifically for N-Myc was successfully established, and N78, the first small molecule inhibitor specifically targeting N-Myc was discovered. Notably, N78 showed a specific affinity for binding to N-Myc, leading to the degradation of N-Myc, inhibition of target gene expression, and a consequential reduction in the viability of N-Myc-dependent tumor cells. Compared to MYCi975, the most promising inhibitor targeting c-Myc/N-Myc, N78 demonstrated acceptable tolerability and significantly more effective tumor regression effects *in vivo*. In terms of the mechanism, N78 specifically promotes the degradation of N-Myc while sparing c-Myc by enhancing the phosphorylation of Threonine-58, which encourages N-Myc ubiquitylation and subsequent destruction. Our research introduces the first selective N-Myc inhibitor, N78, and raises the possibility of using small-molecule N-Myc inhibitors as chemical sensors and potential anti-cancer therapeutics for neuroblastoma.

## Material and Methods

### Chemicals, cell lines and reagents

N78 synthesis was described in supporting information. Human neuroblastoma cell lines, including SK-N-BE(2), IMR-32, SH-SY5Y, SK-N-SH, SUM149PT, MDA-MB-468, MDA-MB-157 and MDA-MB-231 were kindly provided by Stem Cell Bank, Chinese Academy of Sciences. Human neuroblastoma cell line NB1 and human embryonic kidney cell line (HEK293T) were obtained from Meisen Cell Technology Co., LTD. The Aqueous One Solution Cell Proliferation Kit was purchased from Promega CellTiter-Glo (G3581).

### Cell line maintenance

IMR-32, SK-N-SH, SUM149PT and HEK293T were cultured in Dulbecco's modified Eagle's medium; MDA-MB-468, MDA-MB-157 and MDA-MB-231 were cultured in Leibovitz's L-15 Medium. SK-N-BE(2) and SH-SY5Y was cultured in a 1:1 mixture of Dulbecco's Modified Eagle Medium and F12 Medium; NB1 was cultured in a 1:1 mixture of Minimum Essential Medium and RPMI 1640 medium. All culture media were supplemented with 10%-15% fetal bovine serum (FBS) and 1% penicillin-streptomycin. The cell lines were maintained at 37℃ in the presence of 5% CO₂. Routine mycoplasma contamination checks were performed on all cultures.

### Luciferase reporter assay

The full-length *MYCN*, *c-Myc*, and *CREB* genes were cloned in-frame with the GAL4 DNA-binding domain, and the pGAL4-tk-Luc plasmid was utilized as the reporter construct. HEK 293T cells were seeded into 24-well plates and allowed to adhere overnight. The GAL4-*MYCN* or GAL4-*CREB* fusion plasmids, along with the luciferase reporter plasmid (GAL4-tk-Luc) and the Renilla luciferase vector (phRL-TK, Promega), were transiently transfected into HEK 293T cells using Lipofectamine 2000 reagent (Invitrogen). Following a 24-hour transfection period, the cells were treated with the indicated compounds for 12-24 h. Luciferase activities were measured using the Dual-Luciferase Reporter Assay System (Promega), and the results were normalized by calculating the ratio of firefly to Renilla luciferase activity.

### Microscale thermophoresis (MST) assay

The binding affinities of N78 for purified N-Myc and c-Myc were assessed using a Monolith NT.115 instrument (Nanotemper Technologies). N-Myc and c-Myc proteins were labeled with RED fluorescent dye according to the manufacturer's instructions. N78 was prepared in a 2-fold dilution series, resulting in 16 distinct concentrations. The labeled proteins were then mixed with equal volumes of N78 solutions at these varying concentrations. The resulting mixtures were loaded into premium capillaries (NanoTemper Technologies) and measured at 25°C, employing 60% LED power and medium MST power settings. Data were analyzed using MO. Affinity Analysis software (v.2.2.4). All graphical representations were created using GraphPad Prism 7.0.

### Cellular thermal shift assay (CETSA)

For the HEK 293T cell line, transient transfection was performed using the pCDNA3.1-*MYCN* construct for 48 h. Subsequently, the cells were treated with either 10 μM N78 or vehicle (DMSO) for 30 minutes. Following treatment, the HEK 293T cells were harvested using PBS supplemented with the same concentration of compounds or DMSO as used in the initial treatment. The cell suspension was then aliquoted into 0.2 ml PCR tubes (approximately 1 million cells per tube) and subjected to various temperatures for 3 minutes using a PCR instrument (Eppendorf). The freeze-thaw cycle was repeated three times, alternating between liquid nitrogen and 37°C. Finally, the samples were centrifuged, and the supernatant was analyzed by Western blot.

### N-Myc Knockdown

The shRNAs targeting N-Myc are detailed in Table S3. These shRNAs were individually inserted into the pLKO.1 lentiviral vector. The resultant pLKO.1 constructs were co-transfected into HEK 293T cells along with the packaging plasmids pMD2G and pSPAX2 using Lipofectamine 2000 (Thermo Fisher Scientific, Beijing, China). After 72 h of transfection, the harvested lentiviral supernatants were used to infect neuroblastoma cell lines. Stable N-Myc knockdown was selected using puromycin. The knockdown efficiency was validated by western blot.

### Western blot

Neuroblastoma cells were lysed in RIPA buffer containing 1 mmol/L phenylmethylsulfonyl fluoride (PMSF), a protease inhibitor cocktail, and a phosphatase inhibitor cocktail (Sigma). The lysates were then boiled for 10 minutes in loading buffer (2% SDS, 10% glycerol, 10% β-mercaptoethanol, bromophenol blue, and Tris-HCl, pH 6.8). The lysates were separated by SDS-PAGE on polyacrylamide gels and transferred to nitrocellulose membranes. The membranes were probed with specific primary antibodies followed by secondary antibodies and visualized using the LI-COR Odyssey infrared imaging system (LI-COR Biotechnology, Lincoln, NE) and Enhanced chemiluminescence (ECL).

For MG132 experiments, SK-N-BE(2) and IMR-32 cells were seeded into 6-well plates and treated with 5 μM MG132 for 3 hours, followed by the addition of N78 for an additional 2 hours. Cells were then collected for western blot. For cycloheximide (CHX) chase experiments, SK-N-BE(2) and IMR-32 cells were seeded into 6-well plates and treated with N78 (4 μM) for 3 hours, after which 50 μg/ml CHX was added. Cells were collected at the indicated time points for western blot.

Antibody information is listed in Table S1.

### Real-time quantitative PCR (RT-qPCR)

Total RNA was extracted using TRIzol reagent (Takara, Japan), and 1 μg of total RNA was reverse transcribed into cDNA using the cDNA Reverse Transcription Kit (Takara, Japan) according to the manufacturer's instructions. Subsequently, the cDNA was subjected to quantitative real-time PCR (qPCR) using the SYBR Green Master Mix (Thermo Fisher Scientific). The expression levels of N-Myc, MDM2 and ODC1 in SK-N-BE(2) and IMR-32 cells were validated by qPCR. The relative mRNA expression levels were calculated using the 2-ΔΔCT method and normalized to the internal reference gene β-actin. The sequences of the qPCR primers are listed in Table S2.

### Cell viability

Cell viability was assessed using the CellTiter-Glo Assay (Promega, G3581) according to the manufacturer's protocol. In brief, 1000 or 2000 cells were seeded into each well of a 96-well plate in triplicate and treated with the indicated concentrations of N78 or MYCi975 for 72 h. The CellTiter-Glo reagent was added to each well, and the plates were incubated for 1-4 h at 37℃. The OD values at 490 nm were acquired.

### Colony formation

Neuroblastoma cells were seeded into 6-well plates and allowed to adhere overnight. After attachment, cells were treated with various concentrations of N78 or MYCi975 for 1 week incubation. Following treatment, colonies were fixed with 4% paraformaldehyde (PFA) for 15 minutes at room temperature. The fixed colonies were then stained with 0.2% crystal violet for 10 minutes, ensuring complete coverage of the stain. After staining, the colonies were washed three times with distilled water to remove excess stain. Images of the colonies were captured using a digital camera optimized for high-resolution imaging. The number of colonies was quantified by manual counting.

### Apoptosis assay

Apoptosis was assessed using the Annexin V-FITC Apoptosis Detection Kit (Beyotime, C1062L). Neuroblastoma cells were seeded into 6-well plates and treated with N78 for 48 h. Subsequently, the cells were stained with Annexin V-FITC and propidium iodide (PI) according to the manufacturer's instructions. Apoptotic cells were analyzed by flow cytometry using a FACSCalibur instrument (BD Biosciences).

### TUNEL assay

Neuroblastoma cells were seeded onto gelatin-coated glass coverslips and incubated overnight to allow for cell attachment. Subsequently, cells were treated with N78 for 8 hours. Apoptotic cells were identified using the One Step TUNEL Apoptosis Assay Kit (Beyotime, C1086) according to the manufacturer's instructions. Briefly, cells were fixed with 4% paraformaldehyde for 30 minutes at room temperature, permeabilized with 0.2% Triton X-100, and then stained with the TUNEL reaction mixture containing fluorescein-labeled nucleotides. After incubation for 1 hour at 37°C, cells were washed with PBS and mounted with antifade mounting medium. Apoptotic cells were visualized and imaged using a Leica fluorescence microscope (Leica).

### Neuroblastoma tumor xenograft model

BALB/c-nude, male, 6-8-week-old mice were obtained from the Animal Center of East China Normal University. All animal experimental protocols were approved by the Animal Investigation Committee of the Institute of Biomedical Sciences, East China Normal University. The SK-N-BE(2) xenograft tumor models were established by subcutaneously injecting 5 × 10^6^ SK-N-BE(2) cells suspended in a mixture of PBS and 50% Matrigel into the right flank of BALB/c-nude mice. Treatment commenced when the tumor volume reached approximately 100 mm³. Tumor-bearing BALB/c-nude mice were randomly divided into four groups and administered vehicle, MYCi975 and N78 via intraperitoneal injection. Tumor volume and mouse body weight were assessed twice weekly. Tumor volume was calculated using the formula: tumor volume (V) = length × width² × 0.52. At the end of the experiment, the mice were euthanized by CO_2_. The tumors were removed and prepared for Western blot and immunohistochemical analyses.

### Immunohistochemistry

Tissue sections were subjected to deparaffinization and rehydration through an alcohol gradient, followed by treatment with 3% H₂O₂ in methanol to quench endogenous peroxidase activity. The sections were incubated with primary antibodies specific for N-Myc, N-Myc-pT58, and Ki67 overnight at 4°C. On the following day, sections were incubated with secondary antibodies for 1 h. The immuno-reactive signals were detected using the avidin-biotin complex (ABC) system and visualized with 3,3'-diaminobenzidine (DAB) as the substrate. Images were captured using a Leica photomicroscope, and the intensity of immunohistochemical (IHC) staining was quantified using Aperio ImageScope software.

### Hematoxylin and eosin staining

Tissue sections were subjected to deparaffinization and rehydration through a graded ethanol series, followed by standard hematoxylin and eosin (H&E) staining. After staining, the sections were dehydrated through an ethanol gradient and mounted with a resin-based mounting medium. Images were acquired using a Leica photomicroscope, and the histological features were quantitatively assessed using Aperio ImageScope software.

### Statistical analysis

Experiments were carried out with three or more replicates. Student's T-tests did statistical analyses. P values < 0.05 were considered significant. One-way ANOVA determined the differences between control and experimental groups. Data were expressed as means, and 95% confidence intervals and P < 0.05 was considered significant. All analyses were performed using Microsoft Excel 2016 and GraphPad Prism 7.0.

## Results

### N78, a promising small-molecule N-Myc inhibitor

**Figure 1:**
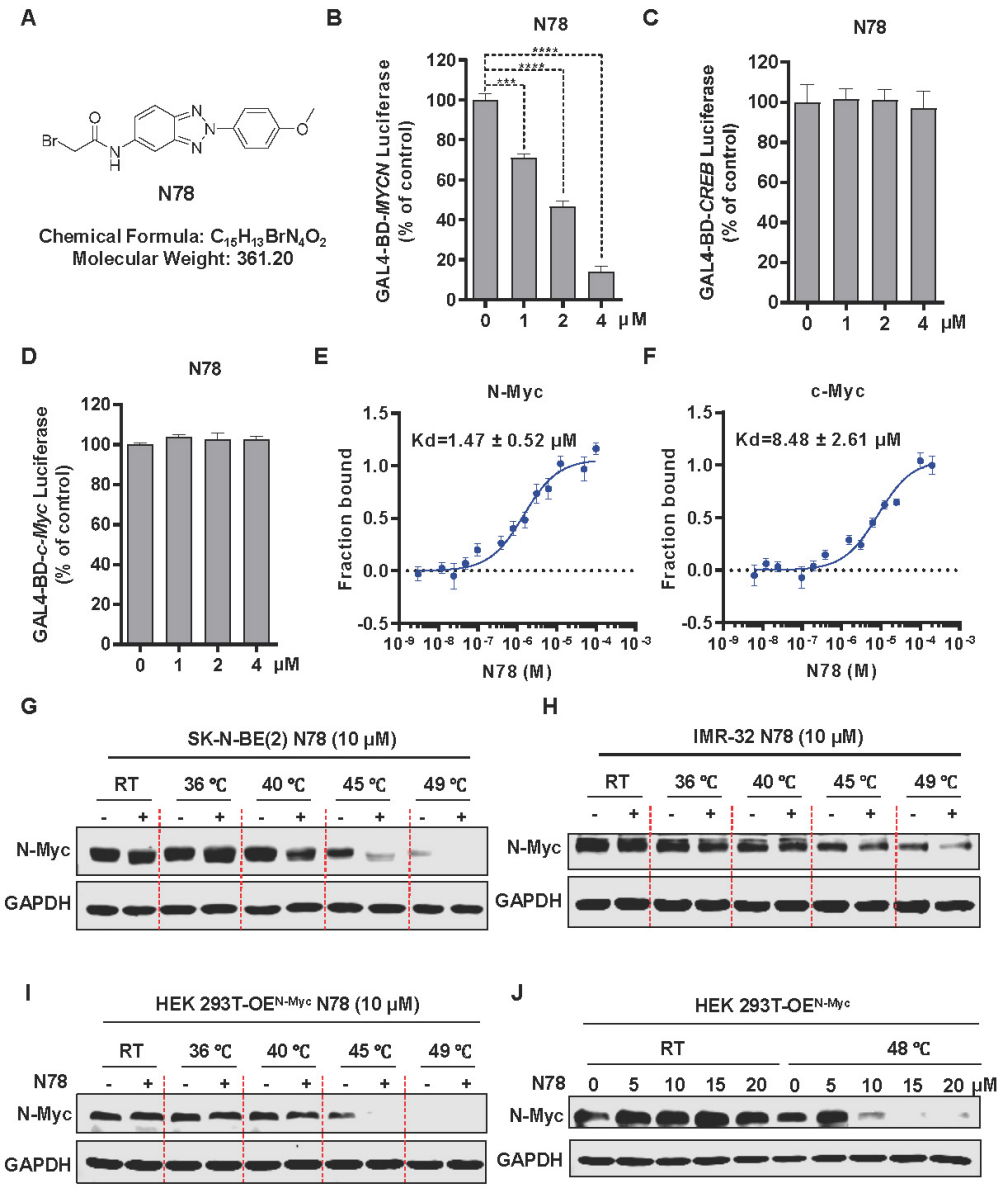
** N78, a promising small-molecule N-Myc inhibitor. (A)** The chemical structure of N78. **(B-D)** Inhibitory activities of N78 on *MYCN, CREB* or *c-MYC* luciferase reporter gene assay. 293T cells were transiently transfected with GAL4-*N-Myc* **(B)**, GAL4-*CREB* **(C)**, or GAL4-*c-Myc* **(D)**, along with GAL4-luciferase and Renilla plasmids. The cells were subsequently treated with different concentrations of N78 for 12 h, then, luciferase activities were assessed. The results were presented as the ratio of firefly to Renilla luciferase activity (n = 3).** (E-F)** MST assay detected binding affinities between N78 and N-Myc or c-Myc (n = 3). N-Myc^1-464^ and c-Myc^1-439^ were fluorescently labeled in MST buffer, mixed with the same volume of unlabeled N78 at 16 different serially diluted concentrations which started at 100 μM or 200 μM, respectively. **(G-H)** Cellular thermal shift assay (CETSA) of N-Myc with N78 treatment in SK-N-BE(2) and IMR-32. SK-N-BE(2) and IMR-32 cells were treated with 10 μM N78 or DMSO. After incubating for 30 min, the cells were divided into five equal portions and lysed for PCR at various temperature gradients (RT, 36°C, 40°C, 45°C, 49°C) and then analyzed by western blotting. **(I)** CETSA of N-Myc with N78 treatment in HEK 293T-OE^N-Myc^. CETSA was performed in the presence of 10 μM N78 or DMSO in 30 min, and the cells were divided into five equal portions and lysed for PCR at various temperature gradients and then analyzed by western blotting. **(J)** CETSA of N-Myc with various concentration N78 treatment in HEK 293T-OE^N-Myc^. Data shown as mean ± sd.

To specifically identify N-Myc inhibitors, we established a screening model targeting N-Myc using a dual-luciferase reporter gene assay. *MYCN*-Gal4BD was overexpressed in 293T cells to specifically activate luciferase expression via a plasmid that carries 5 × Gal4 binding sites. To ensure the specificity of the effects, we used *CREB*-Gal4BD as a negative control. Considering the high homology within the MYC family, we also constructed a *c-Myc-Gal4BD* luciferase assay to assess the selectivity of the compound (Figure S1A). We confirmed the reliability and efficacy of this model through testing with positive controls, including JQ1, a BET inhibitor, and MYCi975, a direct inhibitor of c-Myc/N-Myc^30^, ^31^ (Figure S1B, S1C). Subsequently, through screening our internal small molecule compound chemical library, we identified a promising candidate, N78, which markedly inhibited *MYCN*-Gal4BD luciferase activity at 1 μM, but had no effect on *CREB*-Gal4BD luciferase activity (Figure 1A-1C, Figure S2). At equivalent concentrations, N78 also did not inhibit *c-MYC*-Gal4BD luciferase activity (Figure 1D). To confirm that N78 directly interacted with N-Myc, Microscale Thermophoresis (MST) assays were conducted, showing N78 directly bound N-Myc with *K_D_* of 1.47 ± 0.52 μM (Figure 1E). Simultaneously, the affinity between N78 and recombinant c-Myc protein was 8.48 ± 2.61 µM, nearly six times lower than its affinity for N-Myc (Figure 1F). We also performed the cellular thermal shift assay (CETSA) to examine N-Myc target engagement by N78 in neuroblastoma cells. CETSA measures drug-protein interaction within the protein's native cellular environment, based on ligand-induced changes in protein thermal stability 32. Treatment of SK-N-BE(2) and IMR-32 cells with N78 (10 μM) for 30 min led to significant thermal destabilization (Figure 1G, 1H). Consistent with these results, N78 provoked temperature-dependent and concentration-dependent destabili-zation of N-Myc in 293T cells expressing N-Myc ectopically (Figure 1I, 1J). In summary, we identified and validated a potent and selective N-Myc inhibitor, N78, which specifically targeted N-Myc over c-Myc using a dual-luciferase reporter gene assay, *in vitro* binding experiments, and CETSA in living cells.

### N78 selectively inhibiting cells growth and N-Myc expression of N-Myc-dependent neuroblastoma cells

**Figure 2:**
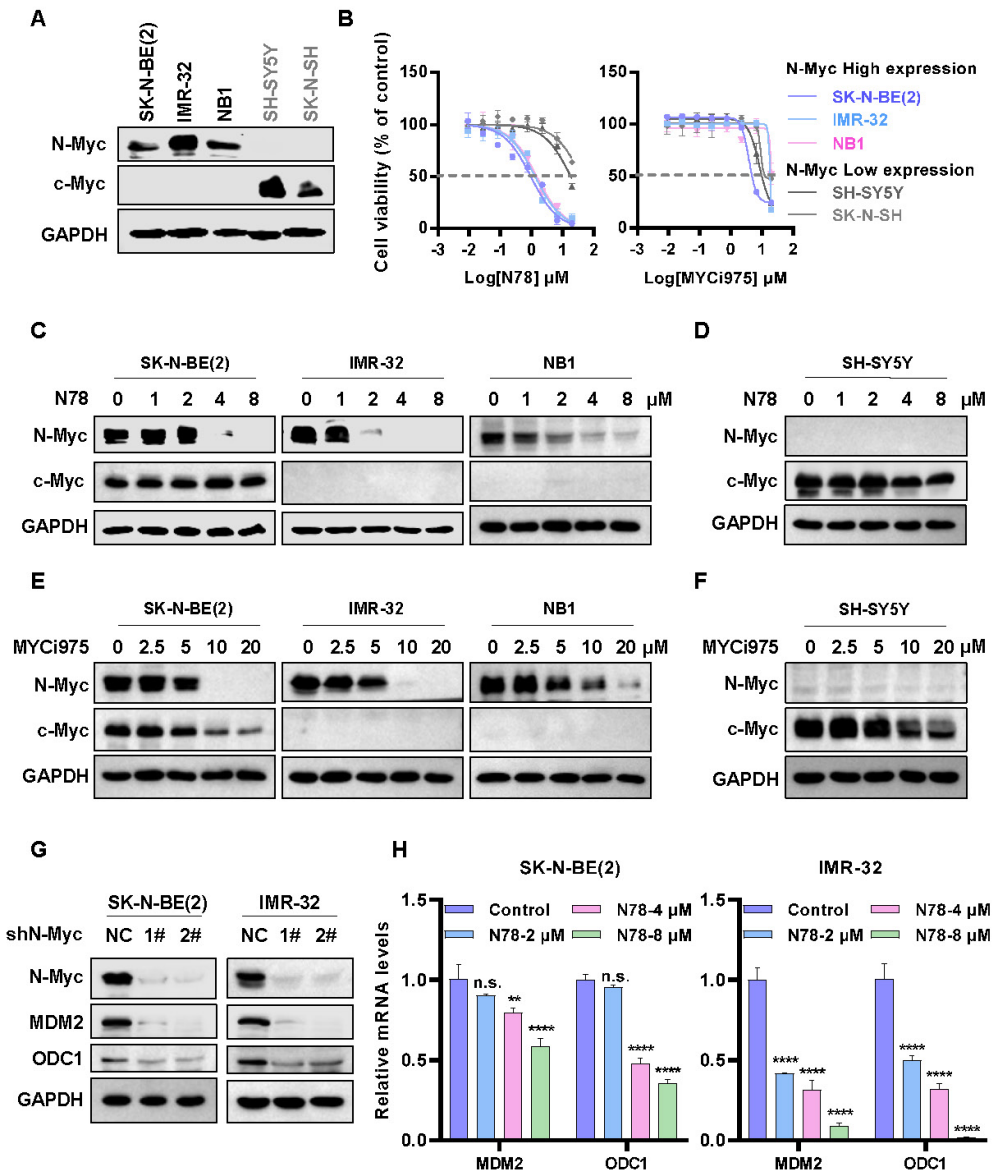
** N78 selectively inhibiting cells growth and N-Myc expression of N-Myc-dependent neuroblastoma cells. (A)** N-Myc and c-Myc expression of different neuroblastoma cell lines (protein loading: 30 μg total protein). **(B)** N78 and MYCi975 inhibited NB cells growth. NB cell lines were plated in 96-well plates with 1000 or 2000 per well and treated with indicated concentrations of N78 or MYCi975 for 72 h. Cell viability was assessed using the MTS assay (n = 3).** (C-D)** N78 selectively inhibited N-Myc expression. SK-N-BE(2), IMR-32, NB1, and SH-SY5Y cells were exposed to different concentrations of N78 for 12 h, then, cell lysates were collected and analyzed by western blot (protein loading: N-Myc/GAPDH, 30 μg total protein; c-Myc, 120 μg total protein). **(E-F)** MYCi975 inhibited N-Myc and c-Myc expression. SK-N-BE(2), IMR-32, NB1, and SH-SY5Y cells were exposed to different concentrations of MYCi975 for 24 h, then, cell lysates were collected and analyzed by western blot (protein loading: N-Myc/GAPDH, 30 μg total protein; c-Myc, 120 μg total protein). **(G)** Knockdown N-Myc inhibited N-Myc downstream gene expression in SK-N-BE(2) and IMR-32. SK-N-BE(2) and IMR-32 cells were transfected with a control shRNA or two independent shRNAs targeting N-Myc, and the expression levels of N-Myc and its downstream genes were analyzed by western blot. **(H)** N78 inhibited N-Myc downstream gene expression in SK-N-BE(2) and IMR-32. Real-time PCR was used to assess the expression of N-Myc downstream gene in SK-N-BE(2) and IMR-32 cells treated with a gradient of N78 concentrations. Data shown as mean ± sd. n.s., not significant, ** P < 0.01, **** P < 0.0001 by one-way ANOVA followed by multiple-comparison tests.

The expression patterns of N-Myc and c-Myc in various neuroblastoma cell lines exhibited distinct characteristics. Specifically, SK-N-BE(2), IMR-32, and NB1 cells demonstrated a significantly high expression of N-Myc, while SK-N-SH and SH-SY5Y showed elevated levels of c-Myc (Figure 2A). High sensitivity to N78 was observed in cell lines with high N-Myc expression, not in those with low N-Myc levels, in both short-term and long-term assays (Figure 2B, Figure S3A, S3C, S3E). Notably, the activity of N78 was superior to that of MYCi975 (Figure 2B, Figure S3B, S3D, S3F). Additionally, N78 selectively inhibited N-Myc protein expression at 1-2 µM in N-Myc-dependent neuroblastoma cells, while showing no significant impact on c-Myc protein expression (Figure 2C, 2D). Compared to MYCi975, N78 demonstrated significantly greater inhibitory activity and selectivity (Figure 2E, 2F). Similarly, treatment of 293T cells overexpressing exogenous *MYCN* and *c-Myc* with N78 and MYCi975 demonstrated that N78 selectively inhibited N-Myc expression, with greater selectivity and potency than MYCi975 (Figure S4). To further validate the selectivity of N78 for N-Myc and c-Myc, we also assessed its antiproliferative activity in c-Myc-addicted triple-negative breast cancer (TNBC) cells. The results indicated that N78 exhibited 3- to 6-fold lower antiproliferative activity in triple-negative breast cancer cells compared with N-Myc-dependent neuroblastoma cells. While, MYCi975 maintained consistent antiproliferative activity across these cell types (Figure S5A-S5D). Consistently, N78 was unable to effectively suppress c-Myc expression in TNBC cells, while MYCi975 demonstrated the inhibitory effects (Figure S5E, S5F). Moreover, the effect of N78 on the direct transcriptional targets of N-Myc, MDM2 and ODC1, was examined. Consistent with N-Myc knockdown, N78 treatment significantly inhibited the expression of MDM2 and ODC1 (Figure 2G, 2H). Taken together, these results demonstrated that N78 suppressed N-Myc-dependent neuroblastoma cancer cell growth, N-Myc and its downstream gene expression, and the anti-proliferative activity of N78 was 3-20 times better than the positive compound MYCi975.

### N78 targets N-Myc inhibiting neuroblastoma cells growth and induces apoptosis

**Figure 3:**
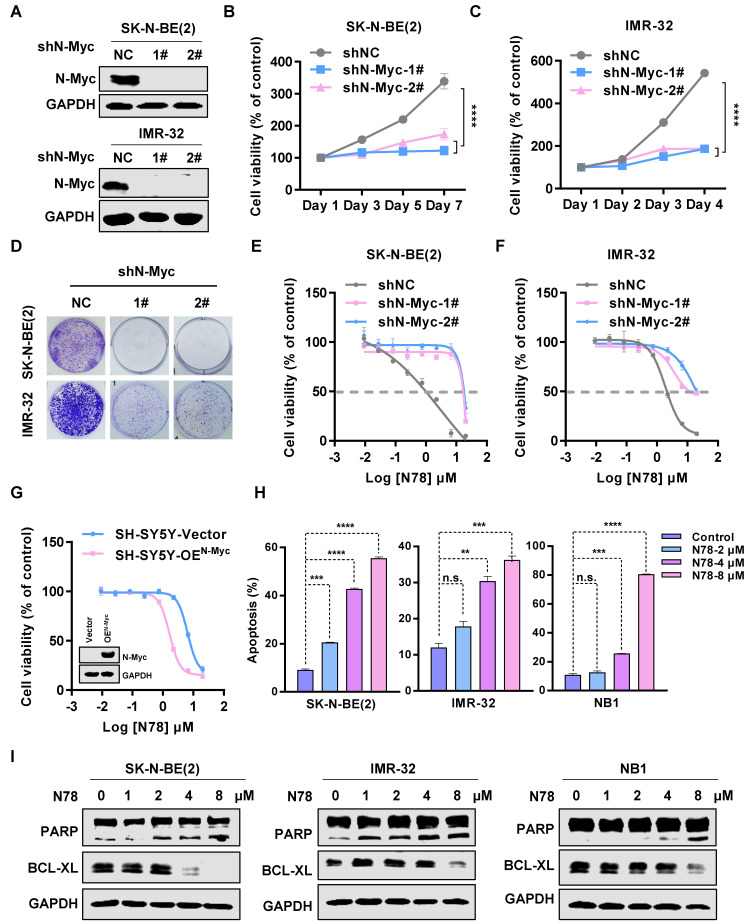
** N78 targets N-Myc inhibiting neuroblastoma cells growth and induces apoptosis. (A)** Knockdown efficacy of N-Myc in NB cell lines was detected by western blot analysis. SK-N-BE(2) and IMR-32 cells were transfected with either a control shRNA or two independent small hairpin RNAs (shRNA) targeting N-Myc, and the expression levels of N-Myc were analyzed by western blot.** (B-C)** N-Myc knockdown inhibited NB cells growth. The different groups of stably transduced with vector control or *MYCN* shRNAs cell viabilities were detected by SRB (n=3). **(D)** Neuroblastoma cells stably transduced with vector control or *MYCN* shRNAs, the colony formation of different groups were detected. **(E-F)** N78 was less effective against the N-Myc knockdown NB cells. The different groups of stably transduced with vector control or *MYCN* shRNAs were treated with indicated concentrations of N78 for 72 h and cell viabilities were detected by MTS (n=3). **(G)** N78 was more sensitive to N-Myc overexpressed SH-SY5Y cell lines (n=3).** (H)** N78 induced apoptosis of NB cells with concentration gradient detected by flow cytometry. The effects of N78 on apoptosis in NB cells (SK-N-BE(2), IMR-32, and NB1) after 48 h treatment were evaluated by flow cytometry. **(I)** N78 induced apoptosis of NB cells with concentration gradient detected by western blot. The effects of N78 on apoptosis in NB cells after 12 h treatment were evaluated by western blot. Data shown as mean ± sd. n.s., not significant, ** P < 0.01, *** P < 0.001, **** P < 0.0001 by one-way ANOVA followed by multiple-comparison tests.

To investigate the biological effects of N-Myc in neuroblastoma cells, we knocked down endogenous N-Myc using shRNA in SK-N-BE(2) and IMR-32 cells (Figure 3A). The knockdown significantly inhibited cell growth and colony formation (Figure 3B-3D). To determine if N-Myc was the primary target of N78, neuroblastoma cells stably expressing N-Myc shRNA or negative control shRNA were treated with N78. In cells with N-Myc knockdown, sensitivity to N78 decreased, while it remained in shNC cells (Figure 3E, 3F). Consistent with these results, ectopic expression of N-Myc in N-Myc low cells (SH-SY5Y) restored their sensitivity to N78 (Figure 3G). Consistent with prior studies, reducing N-Myc expression also triggers apoptosis in neuroblastoma cells (Figure S6A, S6B), we investigated whether N78-induced degradation of N-Myc led to apoptosis 33. Indeed, N78 significantly induced apoptosis in neuroblastoma cells (Figure 3H, Figure S6C). Western blot analysis showed a concentration-dependent increase in cleaved-PARP and a decrease in BCL-XL, indicating upregulation of apoptosis (Figure 3I). Similarly, TUNEL assays confirmed N78 significantly induced apoptosis (Figure S6D). Collectively, this evidence confirms that N78 suppresses neuroblastoma cell growth by targeting N-Myc and inducing apoptosis.

### N78 decreasing N-Myc protein stability by regulating phosphorylation of N-Myc at threonine 58

**Figure 4:**
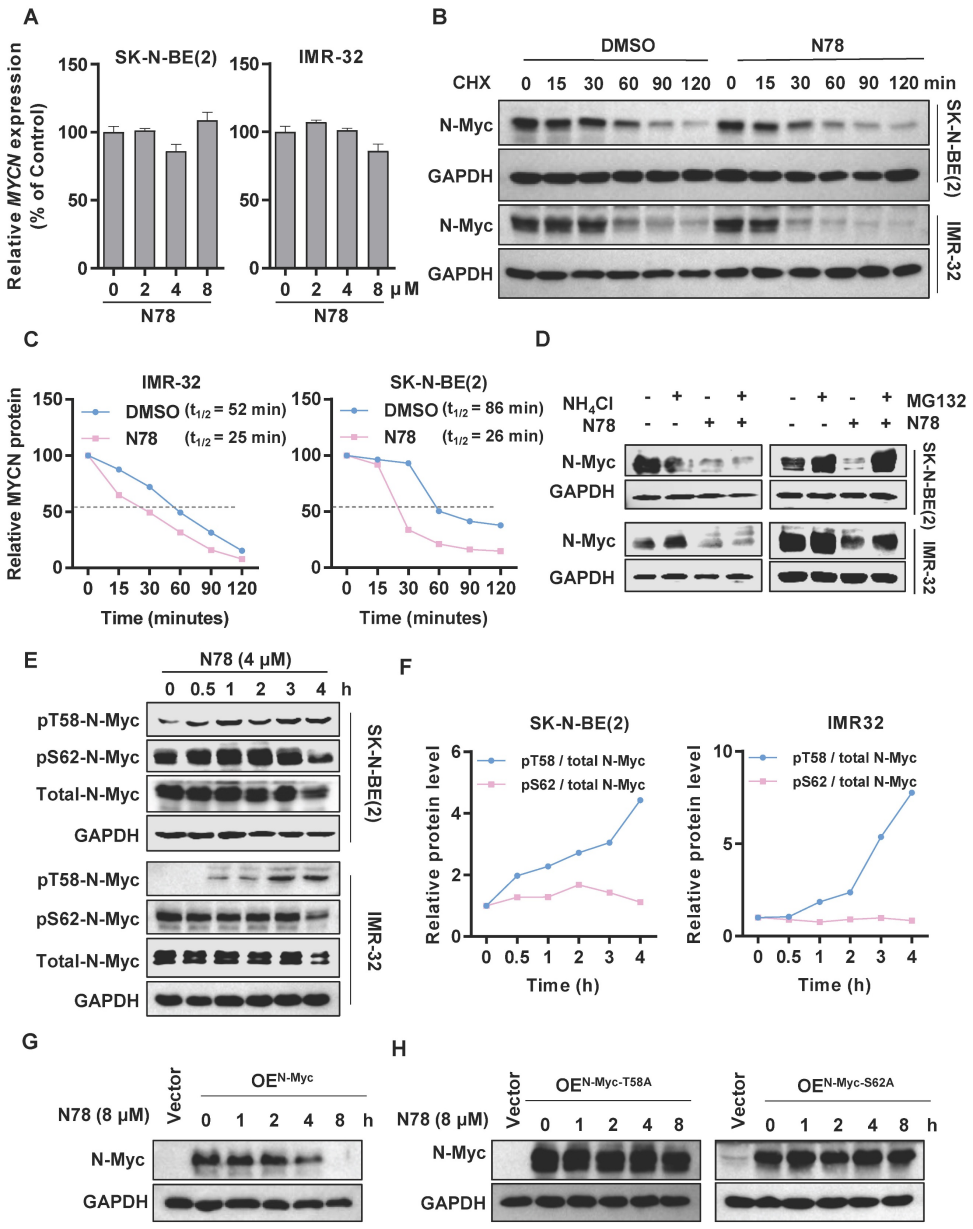
** N78 decreasing N-Myc protein stability by regulating phosphorylation of N-Myc at threonine 58. (A)** RT-qPCR analysis of *MYCN* in SK-N-BE(2) and IMR-32 treatment with N78. The effects of N78 on MYCN mRNA levels after 12 h treatment were evaluated by Real-time PCR. **(B-C)** SK-N-BE(2) and IMR-32 cells were incubated with N78 (4 μM) for 3 h, following by 50 μg/ml cycloheximide (CHX) treatment. N-Myc levels were detected by western blot analysis (B) and the degradation kinetic curves were plotted **(C)**. **(D)** The levels of N-Myc protein under the presence and absence of NH4Cl or N78, MG132 or N78 were determined. SK-N-BE(2) and IMR-32 cells were seeded into 6-well plates and treated with 5 μM MG132 for 3 hours, followed by the addition of N78 (4 μM) for an additional 2 hours. Cells were then collected for western blot. **(E-F)** Phosphorylated N-Myc T58 and S62 in SK-N-BE(2) and IMR-32 treatment with N78 at the indicated time points were detected by western blot **(E)** and the variation curves of pT58 / total N-Myc and pS62 / total N-Myc were plotted (**F**). SK-N-BE(2), IMR-32 cells were exposed to N78 (4 μM) for different time, then, cell lysates were collected and analyzed by western blot. **(G-H)** Western blot of N-Myc in HEK 293T with overexpression of N-My, N-Myc-T58A, N-Myc-S62A after N78 (8 µM) treatment at the indicated time points.

We observed that treatment of neuroblastoma cell lines with N78 reduced N-Myc protein expression, while *MYCN* mRNA levels remained unchanged at the same concentration of N78 (Figure 4A). This suggested that N78 directly reduced N-Myc stability, rather than inhibiting upstream *MYCN* transcription. To confirm the effect of N78 on N-Myc protein stability, we conducted a cycloheximide chase assay, which demonstrated that the half-life of N-Myc protein was significantly reduced in N78-treated cells compared to DMSO-treated cells (Figure 4B, 4C). Investigating the mechanism, we treated SK-N-BE(2) and IMR-32 cells with either NH_4_Cl, a lysosome inhibitor, or MG132, a proteasome inhibitor. We found that N78-induced degradation of N-Myc protein was prevented by MG132 but not NH_4_Cl, indicating that N78 promoted N-Myc degradation through the proteasome pathway (Figure 4D, Figure S7A, S7B). N-Myc protein stability is tightly controlled by various signaling pathways, particularly through an ordered phosphorylation cascade involving sequential phosphorylation of serine 62 (S62) and threonine 58 (T58) by extracellular signal-regulated kinase (ERK, CDK, and JNK) and glycogen synthase kinase 3β (GSK3β), respectively 34. Phosphorylation of threonine 58, recognized by E3 ubiquitin ligases (FBXW7), leads to proteasomal degradation 35. We found that N78 selectively increased phosphorylation at threonine 58 but not at serine 62 of N-Myc-dependent neuroblastoma cells, in a concentration- and time-dependent manner (Figure 4E, 4F, Figure S7C, S7D). Additionally, we determined that phosphorylation of N-Myc at threonine-58 (T58) is essential for N78-induced N-Myc degradation. The N-Myc T58A (threonine-to-alanine) mutant, which could not be phosphorylated at this site, is resistant to degradation by N78 (8 µM). Similarly, the N-Myc S62A (Serine-to-alanine) mutant, which could not be phosphorylated on S62 and thus could not be recognized and phosphorylated on T58 by GSK3β, was also resistant to degradation (Figure 4G, 4H). In summary, N78 treatment enhanced phosphorylation of threonine 58 in neuroblastoma cell lines, leading to subsequent degradation via the proteasome pathway.

### N78 blocking neuroblastoma growth *in vivo*

**Figure 5:**
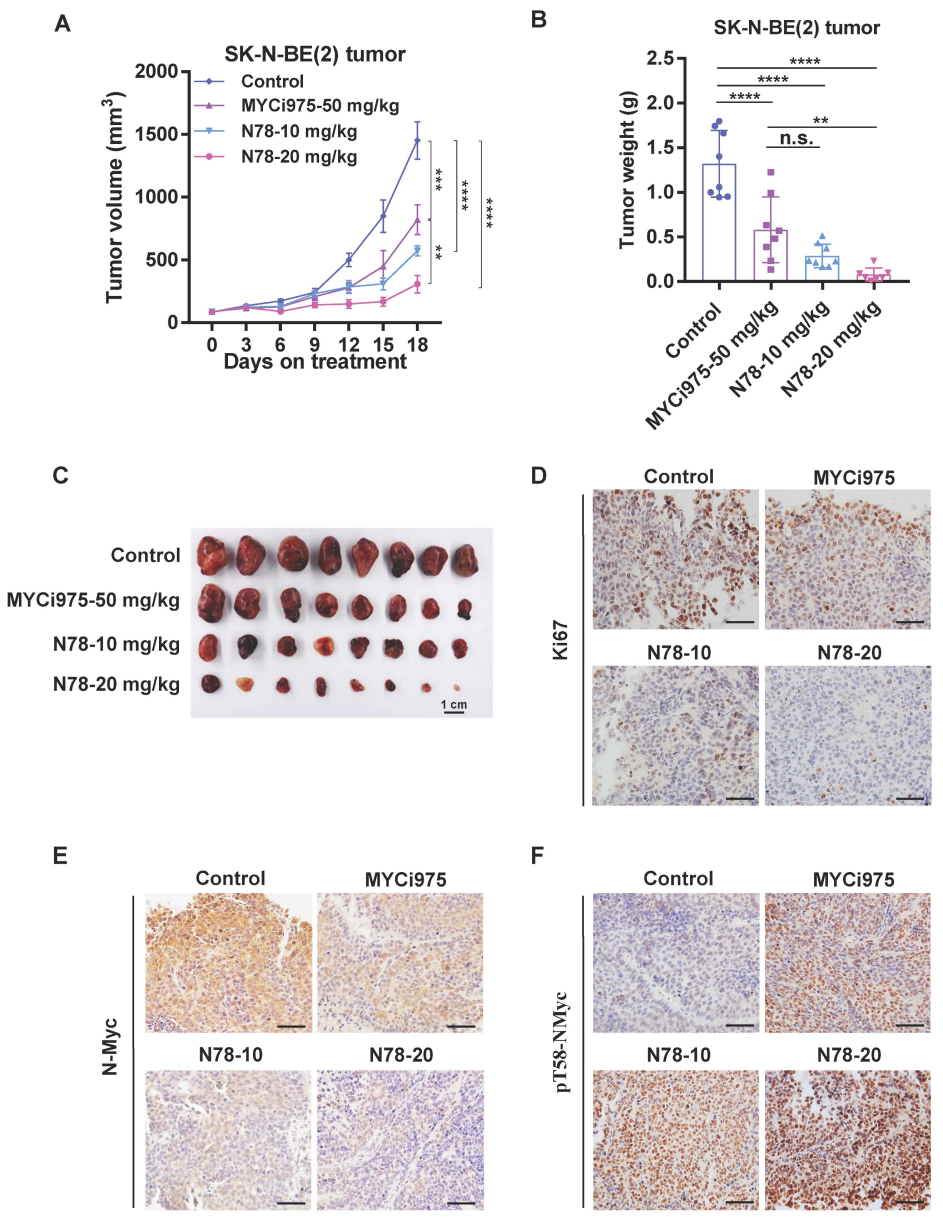
** N78 blocking neuroblastoma growth *in vivo*. (A-C)** SK-N-BE(2) tumor volume were recorded every 3 days (n=8 per group). SK-N-BE(2) cells were suspended in 0.1 mL 50% Matrigel and injected into the right flank of BALB/c-nude. Mice were randomly assigned and i.p. injected with vehicle, MYCi975-50 mg/kg, N78-10 mg/kg and N78-20 mg/kg. Tumor volume **(A)** was measured every 3 days and tumors were stripped, weighed **(B)** and photoed **(C)** at the end of the experiment. **(D-F)** Ki67, N-Myc and phosphorylated N-Myc T58 immunostaining and representative images of SK-N-BE(2) tumors. Scale bar, 50 μm. The immunostaining was quantified using the Aperio ImageScope. Data shown as mean ± sd. n.s., not significant, ** P < 0.01, *** P < 0.001, **** P < 0.0001 by one-way ANOVA followed by multiple-comparison tests.

Building on the potent *in vitro* effects of N78 against N-Myc-dependent neuroblastoma cell lines, we evaluated its therapeutic efficacy *in vivo*. In the SK-N-BE (2) subcutaneous xenograft model, administration of 10 mg/kg/d and 20 mg/kg/d of N78 significantly suppressed tumor growth with Tumor growth inhibition (TGI) values of 79.4% and 61.4%, respectively, compared to 43.7% for the MYCi975 treatment group (Figure 5A). Tumor weights confirmed that N78 was more effective than MYCi975 in inhibiting neuroblastoma growth (Figure 5B, 5C). Tumor tissues underwent immuno-histochemical analysis, showing decreased levels of Ki67 and N-Myc (Figure 5D, 5E) and increased phosphorylation of N-Myc at threonine 58 in the N78 treatment groups (Figure 5F). Furthermore, N78 was well-tolerated, as evidenced by the insignificant mouse body weight loss (Figure S8A) and lack of apparent toxicity in major organs (Figure S8B, S8C). In conclusion, N78 was well-tolerated in mice and more effectively suppressed neuroblastoma tumor growth *in vivo* than the comparator compound, MYCi975.

## Discussion

Neuroblastoma is the most common high-risk pediatric extracranial solid tumor, with a significant lack of effective treatment options 36-38. N-Myc drives cancer cell proliferation, survival, stem cell-like phenotypes, angiogenesis, and metastasis, making it a promising therapeutic target for neuroblastoma. Despite extensive research, directly targeting N-Myc with small molecules remains challenging 13. Promising inhibitors like OMO-103, a peptide-based inhibitor that completed Phase I trials in 2022, bind to all MYC family members, disrupting their interaction with MAX and suppressing target gene transcription. However, OMO-103 shows limited efficacy as a monotherapy. Another candidate, MYCi975, is a preclinical small molecule that binds the HLH domain of c-Myc/N-Myc, enhancing T58 phosphorylation and thus reducing their stability and transcriptional activity 30. Nevertheless, MYCi975 shows poor inhibitory activity and lacks selectivity for N-Myc over other MYC family proteins. The quest for a potent, selective, and effective direct N-Myc inhibitor continues 39, 40. Here, we present N78, a potent and selective N-Myc inhibitor that demonstrates superior efficacy in suppressing neuroblastoma growth and N-Myc signaling both* in vitro* and* in vivo*. N78 regulates N-Myc protein stability through enhanced phosphorylation of threonine 58 leading to proteasomal degradation. Our findings position N78 as a first-in-class selective N-Myc inhibitor and a potential therapeutic candidate for neuroblastoma.

Despite its strengths, this study has limitations. Although MYC typically functions as a transcription factor regulating multiple gene networks, it is essential to understand how N-Myc inhibitors interfere with N-Myc's chromatin binding and MAX heterodimer formation, impacting target gene expression. While we have demonstrated that N78 binds to N-Myc, it remains to be verified whether N78 disrupts the N-Myc/MAX interaction and its affinity for N-Myc/MAX binding sites. Although N78 significantly inhibited the expression of several target genes, its selective regulation of N-Myc target gene expression is unclear. Future studies should explore the epigenomic effects of N78 to assess its impact on the *MYCN* genome fully and provide evidence supporting combined cancer therapy. Furthermore, N-Myc deregulation is implicated in other cancers like medulloblastoma, neuroendocrine prostate cancer, rhabdomyosarcoma, and AML 41, warranting further investigation into N78's efficacy against these N-Myc-driven malignancies.

## Supplementary Material

Supplementary figures and tables.
