# Supplementary material for: A Small Molecule Selectively Targets N-Myc to Suppress Neuroblastoma Cancer Progression
Source: Int J Biol Sci. 2025 Jul 28;21(11):4895–907. doi: 10.7150/ijbs.97195 (PMC12374823; doi:10.7150/ijbs.97195)
Supplement: Supplementary file 1 — Supplementary figures and tables. [file ijbsv21p4895s1.pdf]

Supplementary Materials for

A Small Molecule Selectively Targets N-Myc To Suppress Neuroblastoma Cancer Progression

Ying Miao[1#], Huang Chen[1#], Yuzhan Li[1], Liting Li[1], Jiangnan Ye [1], Jingwen Zhang[1], Jiayu Wang[1], Haigang Wu[1], Guihong Li[4], Yihua Chen[3*], Zhengfang Yi[1,2*], Mingyao Liu[1*]

Affiliations of authors:

[1] Shanghai Key Laboratory of Regulatory Biology, Institute of Biomedical Sciences and School of Life Sciences, East China Normal University, Shanghai 200241, P.R. China.

[2] Changning Maternity and Infant Health Hospital, East China Normal University, 500 Dong Chuan Rd, Shanghai 200241, China.

[3] School of Pharmaceutical Sciences and Yunnan Key Laboratory of Pharmacology for Natural Products and Yunnan College of Modern Biomedical Industry, Kunming Medical University, Kunming, Yunnan 650500, P. R. China.

[4] Southern Medical University Affiliated Fengxian Hospital, 201499, Shanghai, China.

# These authors contributed equally to this work.

Correspondence to: Zhengfang Yi, Ph.D., Mingyao Liu, Ph.D., Institute of Biomedical Sciences, East China Normal University, 500 Dongchuan Rd, Shanghai 200241, China. Yihua Chen, Ph.D., Yunnan College of Modern Biomedical Industry, Kunming Medical University, Kunming, Yunnan 650500, China. (e-mail: zfyi@bio.ecnu.edu.cn, myliu@bio.ecnu.edu.cn, chenyihua@kmmu.edu.cn. Phone: 86-21-54345016; Fax: 86-21-54344922).

This PDF file includes:

Supplementary Methods;

Supplementary Figures (1-8):

Figure S1. *MYCN*, *c-Myc* and *CREB* luciferase assay validations.
Figure S2. The discovery of N-Myc inhibitors.
Figure S3. N78 selectively inhibited exogenous N-Myc expression.
Figure S4. N78 inhibited MYCN-dependent neuroblastoma cancer cell viability.
Figure S5. The effects of N78 and MYCi975 on inhibiting triple-negative breast cancer cell viability.
Figure S6. N78 significantly induced apoptosis.
Figure S7. N78 decreasing N-Myc protein stability by regulating N-Myc-threonine 58 phosphorylation.
Figure S8. N78 was well-tolerated and caused regression of neuroblastoma tumors *in vivo*.

Supplementary Tables (1-3):

Table S1. List of antibodies used and their technical information.
Table S2. The sequence of Real-time PCR primers.
Table S3. The sequence of shRNAs for silencing MYCN.

Supplementary methods

Chemicals

Synthetic scheme of 2-bromo-*N*-(2-(4-methoxyphenyl)-2*H*-benzo[*d*][1,2,3]triazol-5-yl)acetamide (N78)

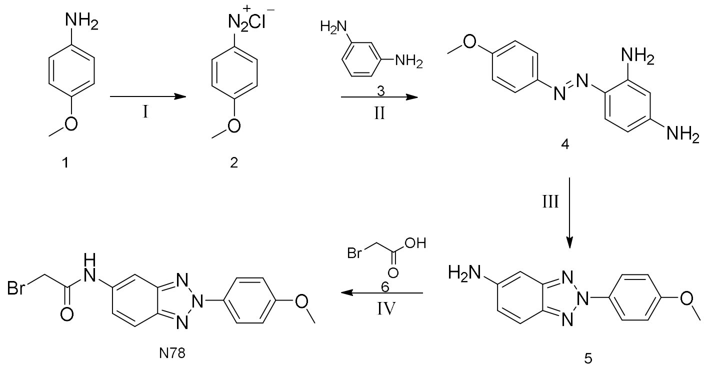

Reagents and conditions: (I) $NaNO_2$, HCl, MeOH, 0 °C; (II) **3**, NaOH, MeOH/H_2O, 0 °C to r.t; (III) $CuSO_4$, NH_4OH, EtOH, H_2O, 100 °C; (IV) **6**, HOBT, EDC·HCl, DMF.

(I) Compound **1** (3.0 g, 24 mmol, 1 eq) was dissolved in MeOH (15 mL), and the reaction mixture was stirred at 0 °C for 10 min, added the HCl (6 mL, 12 mol/L) slowly, then added the $NaNO_2$(3.4 g, 49 mmol, 2 eq). The mixture was stirred at 0 °C for 15 min to afford Intermediate **2**;

(II) Compound **3** (3.2 g, 18 mmol, 1 eq) was dissolved in MeOH (10 mL)/H_2O (5 mL). NaOH (2.7 g, 67 mmol, 4 eq) was added into the solution, then the mixture was added into the Intermediate **2**. After 30 min, the crude product was filtered with water to afford Intermediate **4**.

(III) The mixture of Intermediate **4**, $CuSO_4$ (36 g, 14 mmol, 6 eq), NH_4OH (18 g, 51 mmol, 3 eq) and EtOH (20 mL)/ H_2O (20 mL) was heated at 100 °C for 10 h. The product was filtered and the crude residue was purified by column chromatography to give Intermediate **5**.

(IV) The solution of Intermediate **5** (1.8 g, 7.5 mmol, 1 eq), compound **6** (1.2 g, 8.2 mmol, 1.1 eq) and EDC • HCl (4.4 g, 9.7 mmol, 1.3 eq) in DMF (10 mL) was stirred, then added the HOBT into the mixture. The product was filtered with water, then the crude residue was purified by column chromatography to afford desired compound **N78** (2-bromo-*N*-(2-(4-methoxyphenyl)-2*H*-benzo[*d*][1,2,3]triazol-5-yl)acetamide).

^1H NMR (500 MHz, DMSO-d_6) δ 10.61 (s, 1H), 8.43 (d, J = 1.8 Hz, 1H), 8.24 – 8.19 (m, 2H), 7.99 (d, J = 9.1 Hz, 1H), 7.52 – 7.46 (m, 1H), 7.22 – 7.16 (m, 2H), 4.34 (s, 2H), 3.86 (s, 3H). ^{13}C NMR (125 MHz, DMSO) δ 165.28, 159.78, 144.55, 141.51, 137.33, 133.02, 122.20, 121.47 (2C), 118.60, 114.83 (2C), 104.72, 55.57, 30.33. HRMS (ESI): calculated for $C_{15}H_{13}BrN_4NaO_2$ [M + Na] $^+$: 383.0110, found 383.0114.

Luciferase reporter assay.

Full length MYCN or CREB was cloned in frame to the GAL4 DNA binding domain and pGAL4-tk-Luc was used as reporter constructs. The HEK293T cells were seeded into 24-well plates for overnight, and the GAL4-MYCN or GAL4-CREB fusion plasmids, luciferase reporter plasmids (GAL4-tk-Luc) and Renilla luciferase vector (phRL-TK, Promega) were transiently transfected into HEK293T cells using Lipofectamine 2000 reagent (Invitrogen). After transfection for 24 h, the transfected cells were treated with indicated compounds for 16-20 h. Renilla and firefly activities were determined by luminometry using the Dual-Luciferase Reporter Assay System (Promega) and the ratio calculated. Results were expressed as the ratio of firefly to Renilla luciferase activity.

Hematoxylin and Eosin stain.

Tissue samples were dewaxed and then washed with alcohol gradient, followed by hematoxylin and eosin stain. Then, the samples were dehydrated and mounted with resinous mounting medium. Images were acquired by Leica photomicroscope. And the images were quantified using the Aperio ImageScope.

Supplementary Figures

Miao *et al.* Figure S1

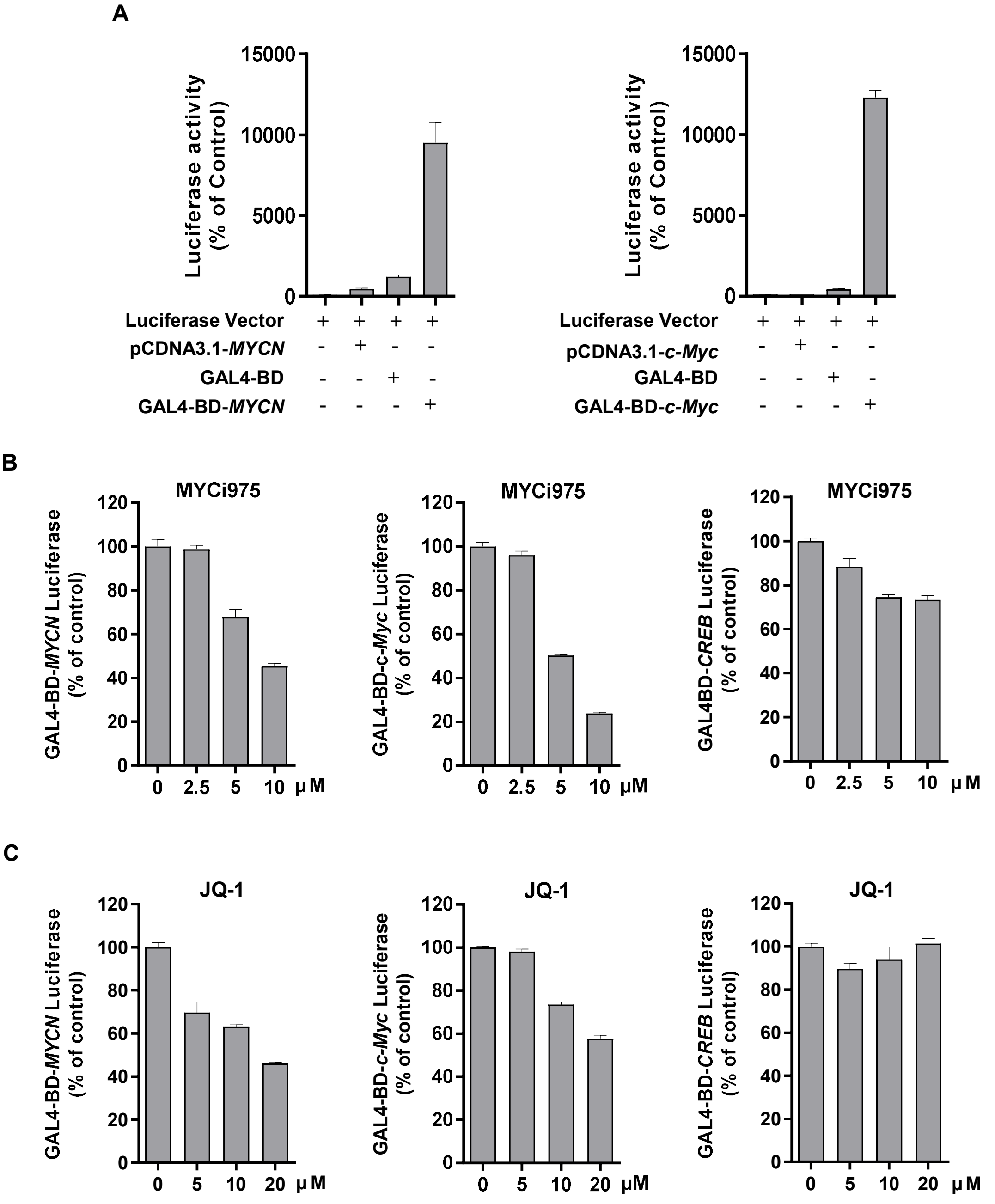

Figure S1. *MYCN*, *c-Myc* and *CREB* luciferase assay validations. (A) Plasmids were transiently transfected into HEK 293T with the indicated groups and luciferase was measured as materials and methods described. **(B-C)** The inhibitory effects of MYCi975 and JQ-1 on the luciferase activities of GAL4-*MYCN*, GAL4-*c-Myc*, and GAL4-*CREB* luciferase reporter gene assays were assessed. The GAL4-*MYCN,* GAL4-*c-Myc* or GAL4-*CREB* fusion plasmids, luciferase reporter plasmids and Renilla plasmids were transiently transfected into HEK 293T for 24 h, and seeded in 24-well plates. MYCi975 (B) and JQ-1(C) were added for another 24 h incubation, and the

luciferase activities was measured as materials and methods described. The results were presented as the ratio of firefly to Renilla luciferase activity (n = 3).

Miao *et al.* Figure S2

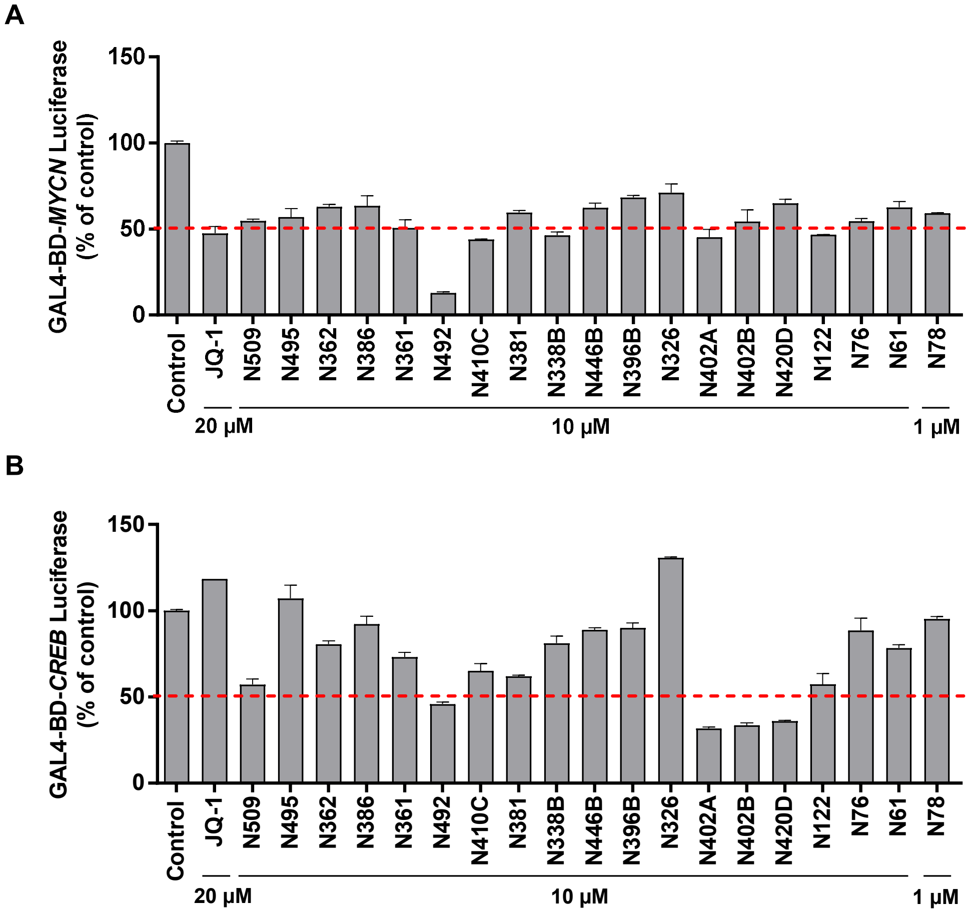

Figure S2. The discovery of N-Myc inhibitors. (A-B) Based on the screening system of GAL4-*MYCN* **(A)** or GAL4-*CREB* **(B)** luciferase, N78 was discovered to significantly inhibit *MYCN*-Luciferase activity with no impact on *CREB*-Luciferase (n=3).

A

N-Myc expression	Cells	IC_{50} (μM) N78
High expression	SK-N-BE(2)	0.95±0.02
	IMR-32	1.31±0.05
	NB1	1.47±0.16
Low expression	SH-SY5Y	18.58±2.60
	SK-N-SH	>20

B

N-Myc expression	Cells	IC_{50} (μM) MYCi975
High expression	SK-N-BE(2)	17.18±0.24
	IMR-32	7.44±0.12
	NB1	>20
Low expression	SH-SY5Y	19.42±0.70
	SK-N-SH	13.37±1.10

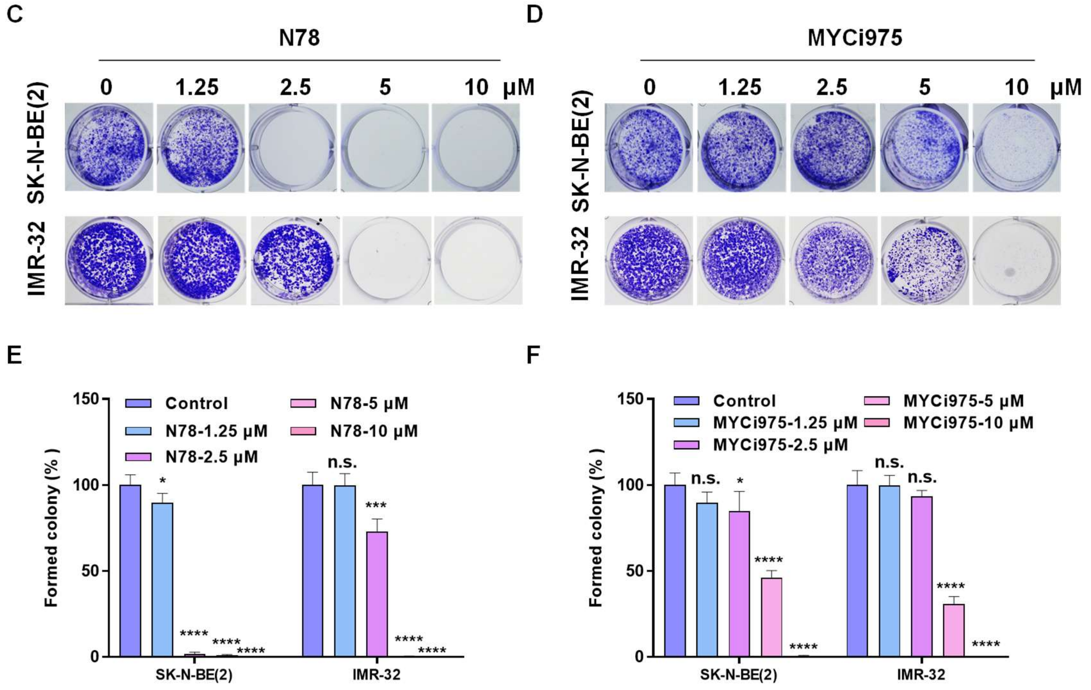

Figure S3. N78 inhibited MYCN-dependent neuroblastoma cancer cell viability. (A-B) N78 and MYCi975 inhibitory activities against neuroblastoma proliferation. Neuroblastoma cell lines with 1000-2000 per well were plated in 96-well plates and treated with indicated concentrations of N78 and MYCi975 for 72 h. Cell viability was assessed using the MTS assay and the IC_{50} values were presented (n = 3). **(C-D)** N78 and MYCi975 inhibited neuroblastoma cancer cells colony formation. Neuroblastoma cancer cells were seeded in 12-well plates, and treated with different doses of N78 and MYCi975 for 1 week and representative images were shown. **(E-F)** Statistical analysis of N78 and MYCi975 inhibiting the colony formation of neuroblastoma cancer cells. Colonies numbers were counted as described in methods (n = 3).

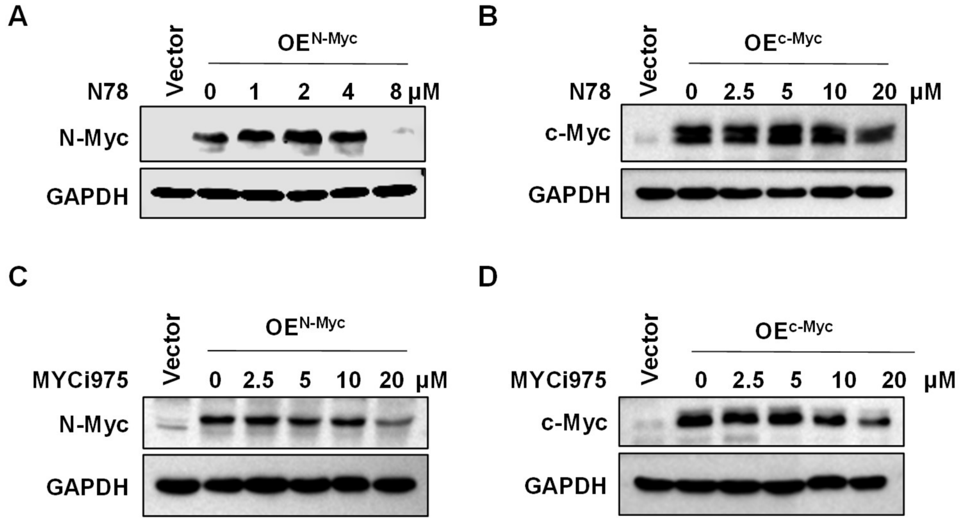

Figure S4. N78 selectively inhibited exogenous N-Myc expression. (A-B) The effects of N78 on inhibiting the expression of exogenous N-Myc and c-Myc were detected by western blot. HEK293T-OE$^{N\text{-}Myc}$ and HEK293T-OE$^{c\text{-}Myc}$ cells were seeded in 6-well plates, different concentrations of N78 were added for 12 h incubation. The inhibition on N-Myc (A) and c-Myc (B) protein expression was detected by western blot. **(C-D)** The effect of MYCi975 on inhibiting the expression of exogenous N-Myc and c-Myc was detected by western blot. HEK293T-OE$^{N\text{-}Myc}$ and HEK293T-OE$^{c\text{-}Myc}$ cells were seeded in 6-well plates, different concentrations of MYCi975 were added for 24 h incubation. The inhibition on N-Myc (C) and c-Myc (D) protein expression was detected by western blot.

A

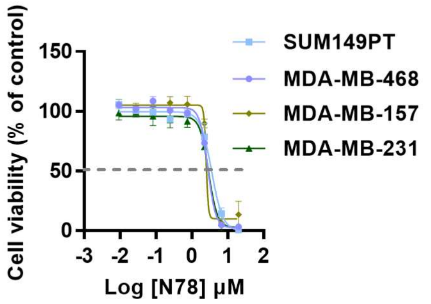

B

	Cells	IC_{50} (μM)
		N78
TNBC	SUM149PT	6.15±1.73
	MDA-MB-468	4.43±0.17
	MDA-MB-157	5.43±0.85
	MDA-MB-231	4.66±0.71

C

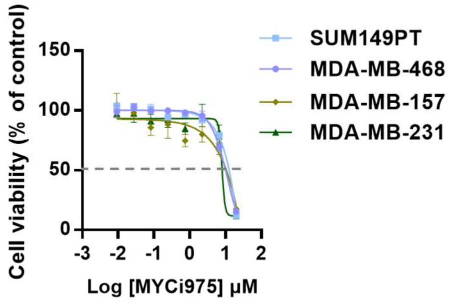

D

	Cells	IC_{50} (μM)
		MYCi975
TNBC	SUM149PT	11.00±0.63
	MDA-MB-468	10.49±0.76
	MDA-MB-157	8.40±1.88
	MDA-MB-231	11.16±2.36

E

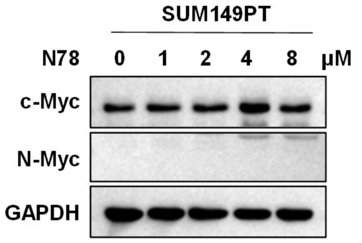

F

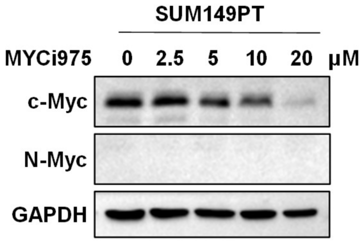

Figure S5. The effects of N78 and MYCi975 on inhibiting triple-negative breast cancer cell viability. (A-B) N78 inhibited triple-negative breast cancer cells growth. TNBC cell lines were plated in 96-well plates with 1000 or 2000 per well and treated with indicated concentrations of N78 for 72 h. Cell viability was assessed using the MTS assay (n = 3). **(C-D)** MYCi975 inhibited triple-negative breast cancer cells growth. Different TNBC cell lines were plated in 96-well plates at optimal densities and treated with indicated concentrations of MYCi975 for 72 h. Cell viability was assessed using the MTS assay (n = 3). **(E)** N78 had no impact on suppressing the expression of c-Myc in the triple-negative breast cancer cell line SUM149PT. SUM149PT cells were exposed to different concentrations of N78 for 12 h, then, cell lysates were collected and analyzed using western blot. **(F)** MYCi975 significantly inhibited the expression of c-Myc in the triple-negative breast cancer cell line SUM149PT. SUM149PT cells were exposed to different concentrations of MYCi975 for 24 h, then, cell lysates were collected and analyzed using western blot.

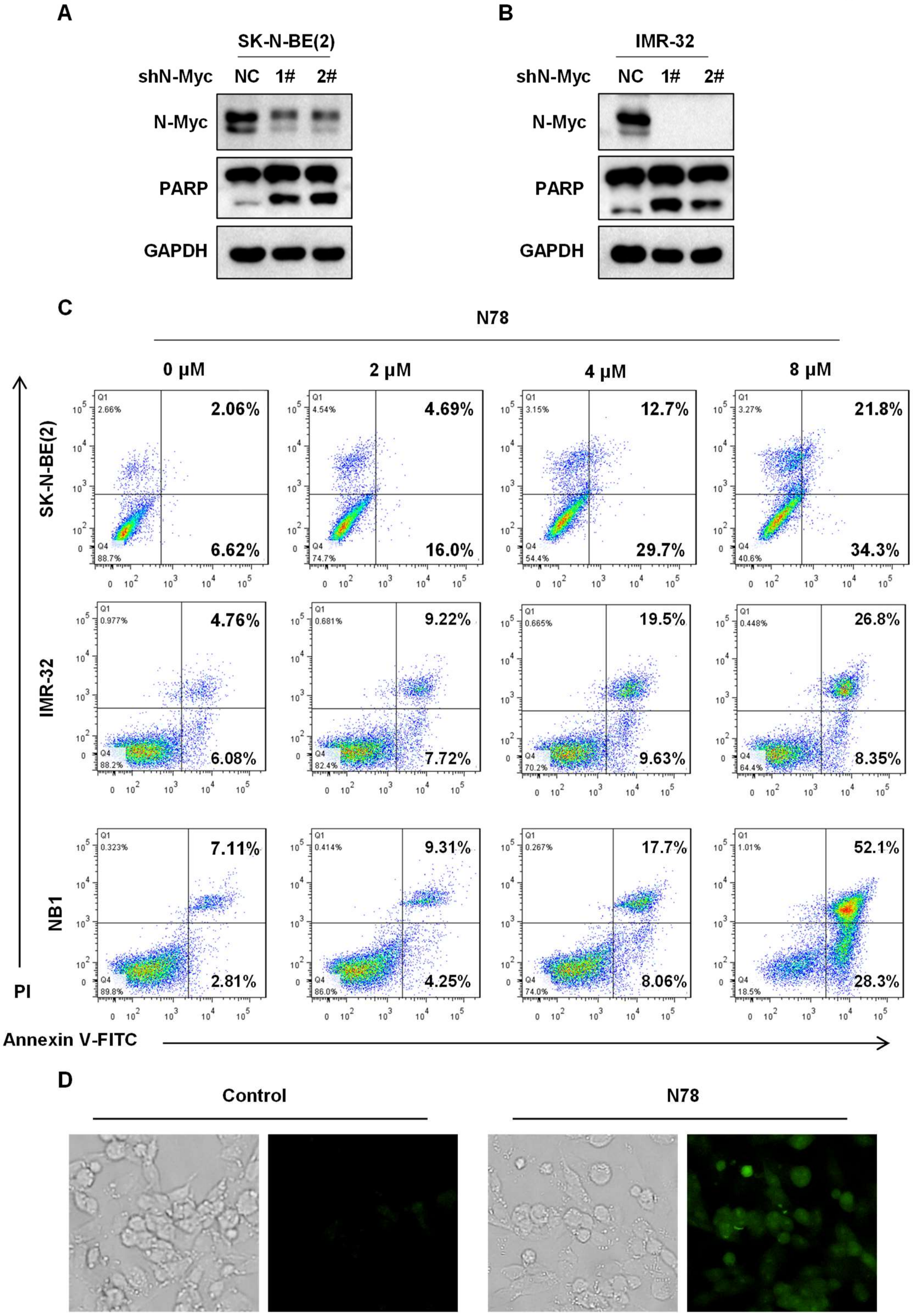

Figure S6. N78 significantly induced apoptosis. (A-B) Knockdown N-Myc induced neuroblastoma cell apoptosis in SK-N-BE(2) and IMR-32. **(C)** Neuroblastoma cancer cells were treated with N78 for 48 h, and apoptotic cells were detected by flow cytometry (n = 3). **(D)** SK-N-BE(2) cells were seeded in climbing sheet and incubated with N78 for 8 hours, followed by staining with TUNEL dye. The photographs were acquired.

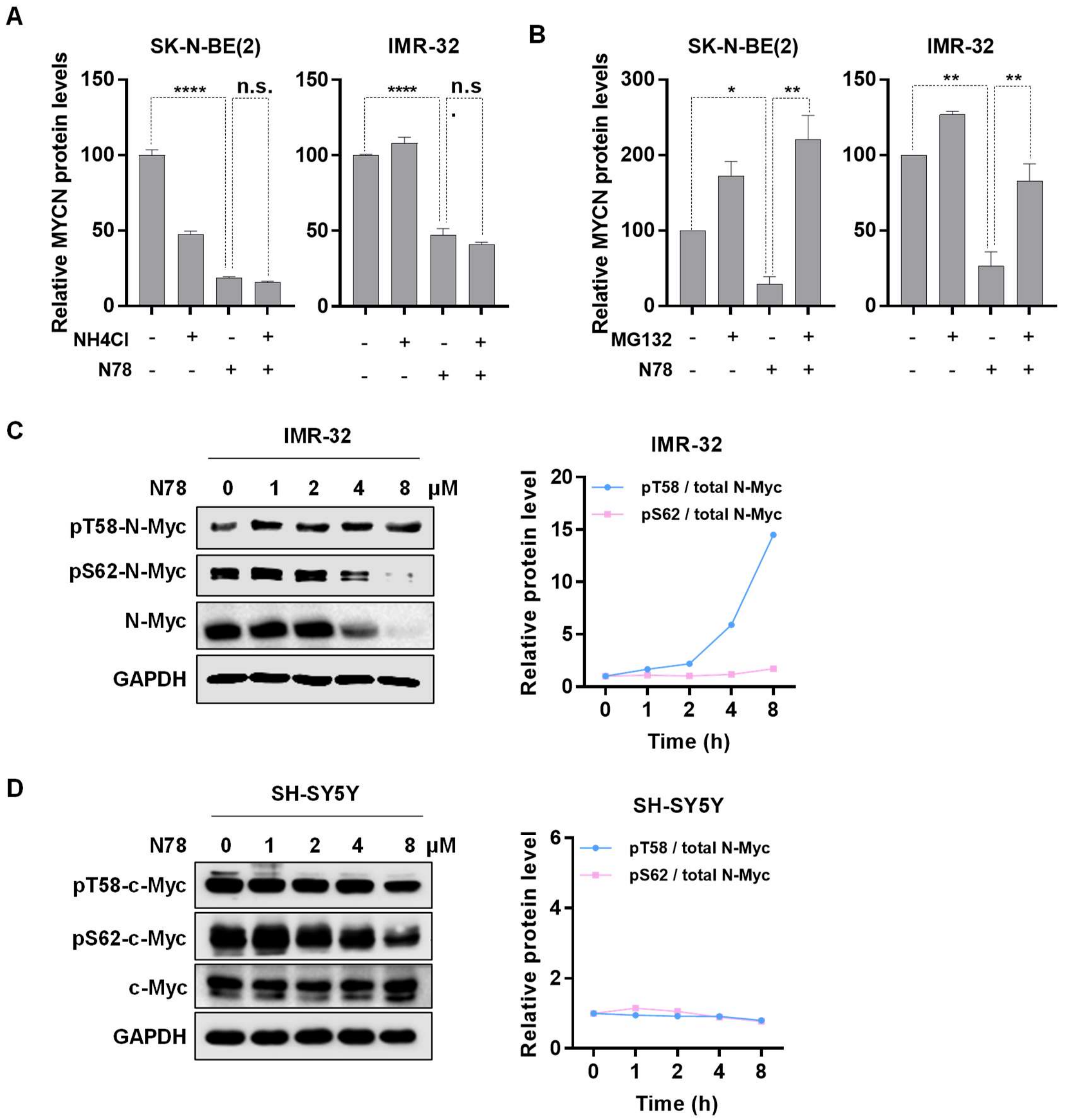

Figure S7. N78 decreasing N-Myc protein stability by regulating N-Myc-threonine 58 phosphorylation. (A-B) The levels of N-Myc protein under the presence and absence of NH4Cl or N78, MG132 or N78 were quantified. SK-N-BE(2) and IMR-32 cells were seeded into 6-well plates and treated with 5 μM MG132 for 3 hours, followed by the addition of N78 (4 μM) for an additional 2 hours. Cells were then collected for western blot. **(C)** Phosphorylated N-Myc T58 and S62 in IMR-32 treatment with N78 at the indicated time points were detected by western blot and the variation curves of pT58 / total N-Myc and pS62 / total N-Myc were plotted. **(D)** Phosphorylated c-Myc T58 and S62 in SH-SY5Y treatment with N78 at the indicated time points were detected by western blot and the variation curves of pT58 / total c-Myc and pS62 / total c-Myc were plotted.

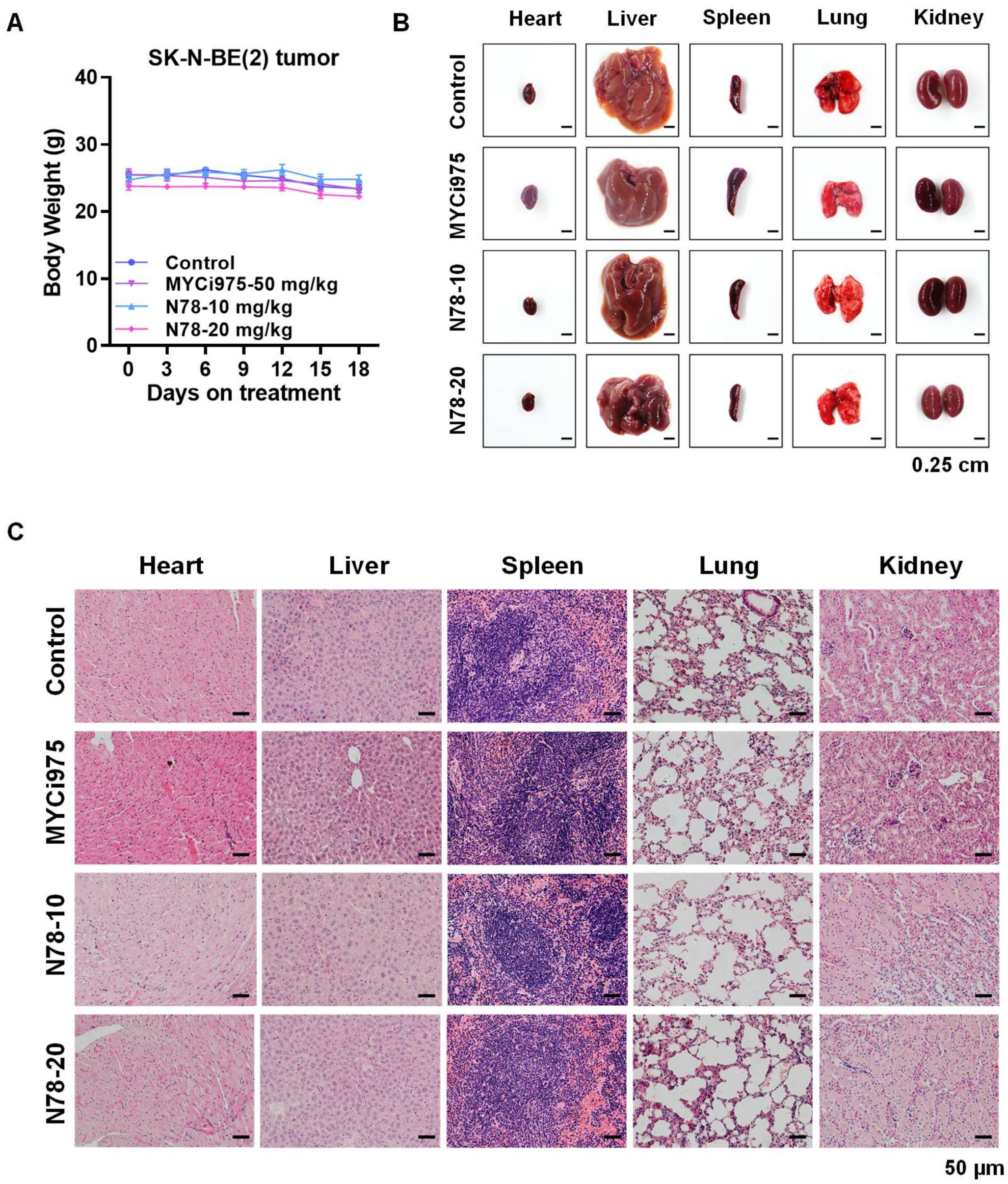

Figure S8. N78 was well-tolerated and caused regression of neuroblastoma tumors *in vivo*. (A) Body weight of different treatment groups of mice was measured every 3 days (n = 8). **(B)** The mice were sacrificed after treatment for 18 days and the organs were excised and photographed. Scale bar, 0.25 cm. **(C)** H&E staining of heart, liver, spleen, lung, kidney tissues of vehicle control and N78 treatment groups. Scale bar, 50 μm. (MYCi975 means MYCi975, 50 mg/kg per day; N78-10 means N78, 10 mg/kg per day; N78-20 means N78, 20 mg/kg per day).

Supplementary Tables

Table S1. List of antibodies used and their technical information

**Primary Antibodies**		
Name	Proteintech	Catalog#
MYCN	CST	10159-2-AP
c-Myc	Abcam	9145
pT58	Abways	CY6356
pS62	Abways	CY5537
cleaved PARP	CST	9548
Bcl-xL	CST	2764
GAPDH	Abcam	ab181602
Ki67	Abcam	ab16667
**Secondary antibodies**		
Name	Source	Catalog#
Goat Anti-Rabbit IRDye 800CW	LI-COR	926-32211
Goat Anti-Mouse IRDye 800CW	LI-COR	926-32210

Table S2. The sequence of Real-time PCR primers

Gene	Forward primer	Reverse primer
β-actin	GTACGCCAACACAGTGCTG	CGTCATACTCCTGCTTGCTG
MDM2	GAATCATCGGACTCAGGTACATC	TCTGTCTCACTAATTGCTCTCCT
ODC1	TTTACTGCCAAGGACATTCTGG	GGAGAGCTTTTAACCACCTCAG

Table S3. The sequence of shRNAs for silencing MYCN

Gene	Sequence (5’ – 3’)
shMYCN-1#	CAGCAGCAGTTGCTAAAGAAA
shMYCN-2#	CTGAGCGATTCAGATGATGAA
shNC	CAACAAGATGAAGAGCACC
